# Prognostic factor of lenvatinib for unresectable hepatocellular carcinoma in real‐world conditions—Multicenter analysis

**DOI:** 10.1002/cam4.2241

**Published:** 2019-05-24

**Authors:** Atsushi Hiraoka, Takashi Kumada, Masanori Atsukawa, Masashi Hirooka, Kunihiko Tsuji, Toru Ishikawa, Koichi Takaguchi, Kazuya Kariyama, Ei Itobayashi, Kazuto Tajiri, Noritomo Shimada, Hiroshi Shibata, Hironori Ochi, Toshifumi Tada, Hidenori Toyoda, Kazuhiro Nouso, Akemi Tsutsui, Takuya Nagano, Norio Itokawa, Korenobu Hayama, Michitaka Imai, Kouji Joko, Yohei Koizumi, Yoichi Hiasa, Kojiro Michitaka, Masatoshi Kudo, the Real‐life Practice Experts for HCC (RELPEC) Study Group, HCC 48 Group (hepatocellular carcinoma experts from 48 clinics in Japan)

**Affiliations:** ^1^ Gastroenterology Center Ehime Prefectural Central Hospital Ehime Japan; ^2^ Department of Gastroenterology and Hepatology Ogaki Municipal Hospital Gifu Japan; ^3^ Division of Gastroenterology and Hepatology, Department of Internal Medicine Nippon Medical School Tokyo Japan; ^4^ Department of Gastroenterology and Metabology Ehime University Graduate School of Medicine Ehime Japan; ^5^ Center of Gastroenterology Teine Keijinkai Hospital Sapporo Japan; ^6^ Department of Gastroenterology Saiseikai Niigata Daini Hospital Niigata Japan; ^7^ Department of Gastroenterology Okayama City Hospital Okayama Japan; ^8^ Department of Hepatology Kagawa Prefectural Central Hospital Takamatsu Japan; ^9^ Department of Gastroenterology Asahi General Hospital Asahi Japan; ^10^ Department of Gastroenterology Toyama University Hospital Toyama Japan; ^11^ Division of Gastroenterology and Hepatology Otakanomori Hospital Kashiwa Japan; ^12^ Department of Gastroenterology Tokushima Prefectural Central Hospital Tokushima Japan; ^13^ Hepato‐biliary Center Matsuyama Red Cross Hospital Matsuyama Japan; ^14^ Faculty of Medicine, Department of Gastroenterology and Hepatology Kindai University Osaka Japan

**Keywords:** adverse event, hand‐foot skin reaction, hepatic function, hepatocellular carcinoma, lenvatinib, modified albumin‐bilirubin grade, prognosis

## Abstract

**Background/aim:**

We assessed suitable factors indicating newly developed lenvatinib (LEN) treatment for unresectable hepatocellular carcinoma (u‐HCC) by investigating real‐world clinical features of patients.

**Materials/methods:**

One hundred fifty two u‐HCC patients, who receive LEN treatment from March to December 2018, were enrolled. (Child‐Pugh score [CPS] 5/6/7/8 = 76/61/13/2, modified albumin‐bilirubin grade [mALBI] 1/2a/2b/3 = 53/35/60/4). Clinical features were evaluated retrospectively.

**Results:**

Overall‐response rate (ORR)/disease control rate (DCR) at 1 month after starting LEN were 38.7%/86.0%, respectively. Estimated median time to progression (TTP) was 7.0 months, while median survival time was not reached within the observation period. CPS (≥7) and past history of tyrosine‐kinase inhibitor (TKI) were not significant prognostic factors. mALBI ≥2b was an only significant prognostic factor (HR 4.632, 95%CI 1.649‐13.02, *P* = 0.004) in Cox‐hazard multivariate analysis. In patients with Child‐Pugh A, c‐index/Akaike's information criterion (AIC) of prognostic predictive value of mALBI were superior to CPS (0.682/135.6 vs 0.652/138.7), while those of stopping LEN also showed that mALBI was better (0.575/447.3 vs 0.562/447.8). Additional analysis of patients with good mALBI (1/2a) revealed that time to stopping LEN was significantly shorter in those with the adverse event (AE) of appetite loss (any grade) than those without (*P* = 0.006) and body mass index (BMI) was also lower in patients with that AE (20.3 ± 3.0 vs 23.6 ± 4.0kg/m^2^, *P* < 0.001), while patients with a hand‐foot skin reaction (any grade) showed good ORR/DCR (59.1%/86.4%) and longer TTP as compared to patients without (*P* = 0.007).

**Conclusion:**

Good hepatic function (mALBI 1/2a) is the best indication for LEN, while potential appetite loss in association with low BMI should be kept in mind in such cases.

## 1 INTRODUCTION

Tyrosine‐kinase inhibitors (TKIs), including sorafenib (SOR)^1^, ^2^ and regorafenib (REG),^3^, ^4^ have been developed for treatment of patients with unresectable hepatocellular carcinoma (u‐HCC). For several years, the only first‐line TKI available for u‐HCC was SOR, though recently lenvatinib (LEN),^5^, ^6^ a newly developed TKI, has become available for first‐line therapy. Unfortunately, serious unmet clinical needs are apparent, as there is no therapeutic option currently available for patients who show intolerability to SOR and/or REG, or failure with REG treatment. In our recent reports of findings obtained in real‐world practice, LEN showed good therapeutic potential not only as a first‐line drug, but also for second‐ and third‐line therapy.^7^, ^8^ Not only TKI naïve but also TKI experienced patients who received the drug showed a similar good therapeutic response,^7^ with similar findings for overall survival (OS) and progression‐free survival (PFS) in each of those groups.^8^ These results revealed that clinical physicians now have a powerful and useful tool in addition to SOR and REG, and the clinical importance and therapeutic efficacy of LEN for u‐HCC is increasingly becoming recognized. Based on these factors, LEN use in clinical practice in Japan is steadily increasing. On the other hand, prognostic factors in u‐HCC patients, who receive LEN treatment, as well as clinical meaning of adverse events (AEs) of its usage have yet to be fully elucidated.

In the present study, we investigated clinical features including hepatic reserve function at the start of LEN therapy along with AEs in patients treated with LEN to elucidate the prognostic factors related to survival and regimen adherence.

## 2 MATERIALS AND METHODS

From March to December 2018, LEN (Lenvima^®^, Eisai Co., Ltd., Tokyo, Japan) was given to 198 patients receiving treatment for u‐HCC at 13 different institutions and hospital groups in Japan (Ehime Prefectural Central Hospital [n = 27], Nippon Medical School Hospital group [Sendagi Hospital, Chiba Hokusoh Hospital, Musashi Kosugi Hospital] [n = 37], Ehime University Hospital [n = 22], Teine Keijinkai Hospital [n = 20], Ogaki Municipal Hospital [n = 18], Saiseikai Niigata Daini Hospital [n = 16], Kagawa Prefectural Central Hospital [n = 14], Okayama City Hospital [n = 13], Asahi General Hospital [n = 12], Toyama University Hospital [n = 6], Otakanomori Hospital [n = 6], Tokushima Prefectural Central Hospital [n = 4], Matsuyama Red Cross Hospital [n = 3]). We examined the records of those patients and collected clinical data obtained at the introduction of LEN. Following exclusion of those who started with a reduced LEN dose, 152 patients were enrolled. Clinical characteristics, prognostic factors related to death or interruption of LEN, and therapeutic response, including time to progression (TTP), OS, and AEs, were analyzed in a retrospective manner. Patients positive for anti‐hepatitis C virus (HCV) were judged to have HCC due to HCV, while those positive for hepatitis B virus surface antigen (HBsAg) were judged to have HCC due to hepatitis B virus (HBV).

### 2.1 Assessment of hepatic reserve function and prognosis

Child‐Pugh score and classification,^9^ and modified albumin‐bilirubin (ALBI) grade (mALBI) 10, 11 were used to assess hepatic reserve function. ALBI score was calculated based on serum albumin and total‐bilirubin values using the following formula: [ALBI‐score = (log10 bilirubin (µmol/L) × 0.66) + (albumin (g/L) × −0.085)], with grading defined as following: ≤−2.60 = ALBI grade 1, >−2.60 to ≤−1.39 = ALBI grade 2, >−1.39 = ALBI grade 3.^12^, ^13^, ^14^ For a more detailed evaluation of hepatic function, we use mALBI, which was done by subdividing the middle ALBI grade 2 into 2a and 2b using the score of −2.270, reported to be the cut‐off value for 30% of indocyanine green retention 15 minutes (ICG‐R15), as the dividing point, for a total of four grades.^10^, ^11^

### 2.2 Diagnosis and treatment of HCC

HCC was diagnosed based on an increasing course of alpha‐fetoprotein (AFP), as well as dynamic CT,^15^ magnetic resonance imaging (MRI),^16^, ^17^ contrast enhanced ultrasonography with perflubutane (Sonazoid^®^, Daiichi Sankyo Co., Ltd. Tokyo, Japan),^18^, ^19^ and/or pathological findings. Tumor node metastasis (TNM) stage proposed by the American Joint Committee on Cancer (AJCC)/Union for International Cancer Control (UICC) was used for evaluation of tumor progression, as well as that previously reported in studies for staging of HCC conducted by the Liver Cancer Study Group of Japan, 6th edition (LCSGJ 6th).^20^

### 2.3 LEN treatment and assessment of AEs

After obtaining written informed consent from each patient, LEN treatment was started. LEN was orally administered at 8 mg/day to patients weighing <60 kg and 12 mg/day to those ≥60 kg, and discontinued when any unacceptable or serious AE or clinical tumor progression was observed. According to the guidelines for administration of LEN, the drug dose was reduced or treatment interrupted when a patient developed any grade 3 or more severe AE, or if any unacceptable grade 2 drug‐related AE occurred. AEs were assessed according to the National Cancer Institute Common Terminology Criteria for Adverse Events, version 4.0.^21^ The worst grade for each AE during the observation period was recorded. If a drug‐related AE occurred, dose reduction or temporary interruption was maintained until the symptom was resolved to grade 1 or 2, according to the guidelines provided by the manufacturer.

### 2.4 Evaluation of therapeutic response

Local physicians at each institution evaluated tumors using enhanced CT or MRI results obtained at 4 or 12 weeks after introducing LEN, in accordance with the modified Response Evaluation Criteria in Solid Tumors (RECIST) guidelines.^22^, ^23^

The present study protocol was approved by the Institutional Ethics Committee of Ehime Prefectural Central Hospital (No. 29‐75). This was a retrospective analysis of records stored in a database and official approval was received based on the Guidelines for Clinical Research issued by the Ministry of Health and Welfare in Japan. All procedures complied with the declaration of Helsinki.

### 2.5 Statistical analysis

Data are expressed as the mean and standard deviation. Statistical analyses were performed using Welch's t‐test, Student's t‐test, Fischer's exact test, Mann‐Whitney's *U* test, Cox hazard analysis, the Kaplan‐Meier method, a log‐rank test, c‐index, Akaike's information criterion, receiver operating characteristic (ROC), and area under the curve (AUC). A *P* value less than 0.05 was considered to indicate statistical significance. All statistical analyses were performed using Easy R (EZR) version 1.29 (Saitama Medical Center, Jichi Medical University, Saitama, Japan),^24^ a graphical user interface for R (The R Foundation for Statistical Computing, Vienna, Austria).

## 3 RESULTS

Clinical characteristics are presented in Table 1. The median age was 71 years and 75.7% were male (n = 115). Average body mass index (BMI) was 22.1 kg/m^2^. Child‐Pugh scores of 5, 6, 7, and 8 were noted in 76, 61, 13, and 2, respectively, while mALBI 1, 2a, 2b, and 3 were seen in 53, 35, 60, and 4, respectively. The median ALBI score was −2.41. According to LCSGJ 6th, TNM stage I, II, III, IVa, and IVb was noted in 1, 21, 52, 12, and 66, respectively. Sixty patients (39.5%) had a past history of SOR treatment, while 16 of those (26.7%) had a history of REG.

**Table 1:** Characteristics of all patients (n = 152)

	n = 152
Age, y^a^ (IQR)	71 (65‐76)
Gender, male:female	115:37
BMI, kg/m^2^ a (IQR)	22.1 (20.7‐24.6)
ECOG PS, 0:1:2	126:23:3
Etiology, HCV:HBV:alcohol:other	65:30:25:32
AST, IU/L^a^ (IQR)	43 (30‐63)
ALT, IU/L^a^ (IQR)	31 (21‐47)
Platelets, x10^4^/µL^a^ (IQR)	13.7 (9.5‐17.0)
Total bilirubin, mg/dL^a^ (IQR)	0.8 (0.2‐1.0)
Albumin, g/dL^a^ (IQR)	3.6 (3.2‐4.0)
Prothrombin, (%)^a^ (IQR)	87 (79‐97)
Child‐Pugh score, 5:6:7:8	76:61:13:2
mALBI grade, 1:2a:2b:3	53:35:60:4
(ALBI score^a^; IQR)	(−2.41, −2.68‐‐1.96)
AFP, ng/mL^a^ (IQR)	42.0 (6.7‐713.1)
Intrahepatic tumor size, cm^a^ (IQR)	3.3 (1.8‐5.2)
Number of intrahepatic tumors, none:single:multiple	17:10:125
TNM stage, LCSGJ 6th, I:II:III:IVa:IVb	1:21:52:12:66
TNM stage, UICC/AICC 8th, IA:IB:II:IIIA:IIIB:IVA:IVB	0:3:54:13:5:15:62
Positive for MVI, Vp1:Vp2:Vp3:Vp4:Vv1:Vv2:Vv3^b^	2:11:5:3:1:4:6
Positive for EHM, LN:lung:bone:peritoneum:adrenal gland:others^b^	23:22:14:10:3:4
Naïve:recurrence	8:144
Past history of hypertension (%)	57 (37.5)
Past history of diabetes mellitus (%)	45 (29.6)
Past history of SOR (%) [REG]	60 (39.5), (REG: 16 [26.7%: 16/60])
Initial dose of LEN, 8:12 mg	87:65
Observation period after starting LEN, days^a^ (IQR)	126 (64‐198)

Abbreviations: IQR: interquartile range, BMI: body mass index, ECOG PS: Eastern Cooperative Oncology Group Performance status, HCV: hepatitis C virus, HBV: hepatitis B virus, AST: aspartate transaminase, ALT: alanine aminotransferase, ALBI score: albumin‐bilirubin score, mALBI: modified ALBI grade, TNM stage: tumor node metastasis stage, LCSGJ 6th: Liver Cancer Study Group of Japan 6th edition, AJCC/UICC 8th: American Joint Committee on Cancer/Union for International Cancer Control, 8th edition, MVI: macrovascular invasion, EHM: extrahepatic metastasis, LN: lymph node, SOR: sorafenib, REG: regorafenib, LEN: lenvatinib

a Median

b Overlapping cases.

**Figure 1:**
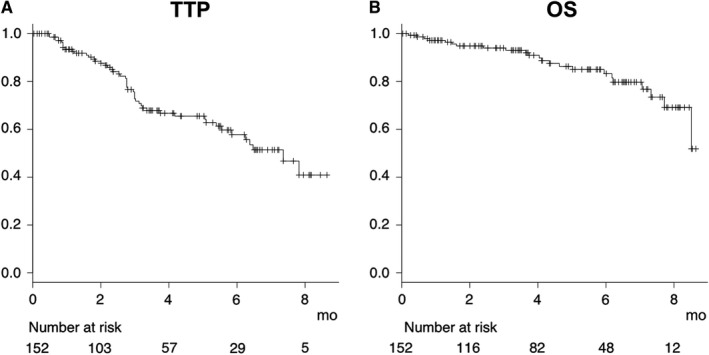
Time to progression (TTP) and overall survival (OS) for all patients (n = 152). The estimated median TTP was 7.0 months (A) and estimated median overall survival time was not reached during the observation period (B)

The median observation period was 126 days. Estimated median TTP was 7.0 months. Estimated median survival time (MST) was not reached within the present observation period (Figure 1). The objective response rate (ORR) at 1 month after starting LEN as shown by mRECIST was 38.7%, while the disease control rate (DCR) was 86.0% (complete response [CR] in three, partial response [PR] in 33, no change [NC], which was non‐CR, non‐PR and non progressive disease [PD], in 44, PD in 13). ORR and DCR at 3 months were 29.4% and 69.4%, respectively (CR, PR, NC, PD; n = 6, 19, 34, 26, respectively). Patients with PD at 1 month (n = 13) showed worse prognosis as compared to the others (CR, PR, NC; n = 80) (MST: 4.5 vs 9.3 months, *P* < 0.001) (Supplemental Figure S1). Prognosis of group of patients with each TNM stage was not different in the present analysis (*P* = 0.226) (Supplemental Figure S2). In addition, after exclusion of patients without the data of best therapeutic response of SOR (n = 5), the therapeutic effect of LEN might be worse in patients with PD (PD) (n = 19) than the others (non‐PD) (n = 36) with regard to therapeutic best‐response of previous SOR treatment (6 months survival rate: 87.5% vs 74.8%, *P* = 0.012) (Supplemental Figure S3).

**Figure 2:**
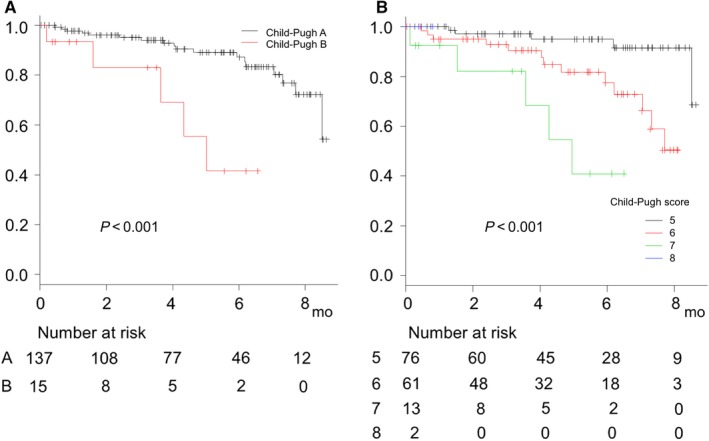
Overall survival of all patients based on Child‐Pugh class and score (n = 152). (A) Overall survival for Child‐Pugh B was worse as compared to Child‐Pugh A (estimated median survival time for Child‐Pugh A not reached during observation period, for B 5.5 months) (*P* < 0.001). (B) Prognosis worsened with worse scores (estimated median survival for Child‐Pugh score 5 and 6, not reached during observation period and for 7 or more 5.5 months, *P* < 0.001)

**Figure 3:**
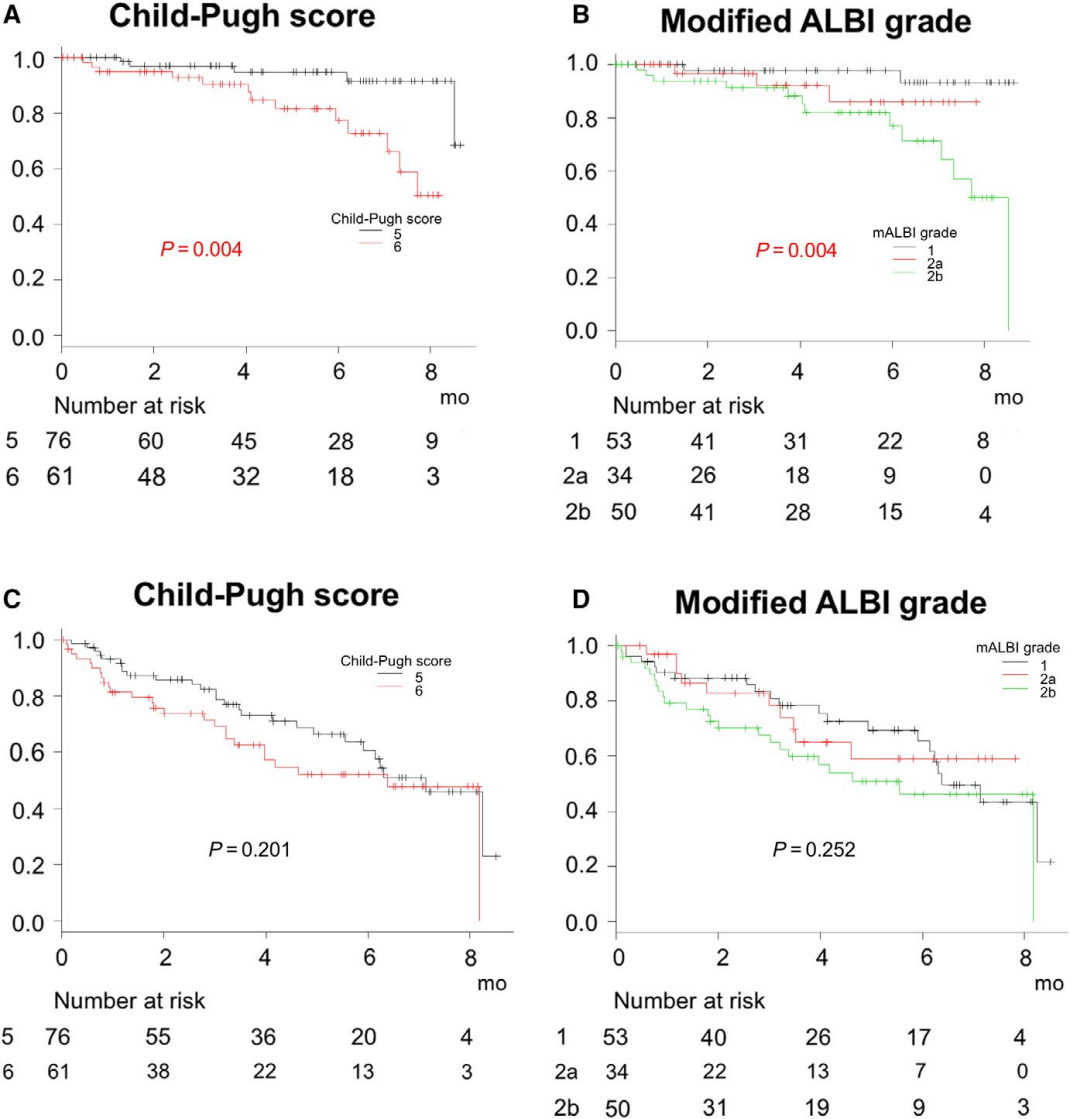
Overall survival of patients with Child‐Pugh A based on Child‐Pugh score and modified ALBI grade (n = 137). (A) Overall survival for Child‐Pugh score 6 was worse as compared to a score of 5 (estimated median survival time for both not reached during observation period) (*P* = 0.004). (B) When prognosis was analyzed using modified ALBI grade (mALBI), prognosis worsened with worse mALBI grade (estimated median survival time for mALBI 1 and 2a not reached during observation period, for 2b 8.4 months) (*P* = 0.004). C‐index and Akaike's information criterion (AIC) predictive values for prognosis based on mALBI grade were better as compared to those based on Child‐Pugh score (0.682 and 135.6 vs 0.652 and 138.7, respectively), and (C, D) the predictive values for time to stopping LEN based on mALBI grade were also superior as compared to those based on Child‐Pugh score (0.575 and 447.3 vs 0.562 and 447.8, respectively)

From the viewpoint of hepatic reserve function, the prognosis of patients with Child‐Pugh B was worse as compared to those with Child‐Pugh A (*P* < 0.001) (Figure 2A). When prognosis was analyzed according to Child‐Pugh score, that worsened with a decline in score (*P* < 0.001) (Figure 2B). Univariate Cox‐hazard analysis of prognostic factors at the time of starting LEN for survival of all patients showed that Child‐Pugh score (≥7) (hazard ratio [HR] 4.998, 95% confidence index [CI] 1.789‐13.96, *P* = 0.002) and mALBI ≥ 2b (HR 5.520, 95%CI 2.042‐14.92, *P* < 0.001) were significant prognostic factors, while multivariate Cox‐hazard analysis showed mALBI ≥ 2b as the only prognostic factor related to death (HR 4.632, 95%CI 1.649‐13.02, *P* = 0.004) Past history of TKIs was not a significant prognostic factor. (Table 2). In patients with Child‐Pugh A, overall survival for Child‐Pugh score 6 was worse as compared to a score of 5 (estimated median survival time for both not reached during observation period, 6‐months survival rate: 91.4% vs 81.7%) (*P* = 0.004). When prognosis was analyzed using modified ALBI grade (mALBI), prognosis worsened with worse mALBI grade, similarly (estimated median survival time for mALBI 1 and 2a not reached during observation period, for 2b 8.4 months) (*P* = 0.004) (Figure 3A,B). However, c‐index and AIC of prognostic predictive value of mALBI were superior to those of Child‐Pugh score (0.682/135.6 vs 0.652/138.7), while those of predictive value for time to stopping LEN were also better than Child‐Pugh score (0.575/447.3 vs 0.562/447.8) (Figure 3C,D). ORR and DCR for patients with mALBI 1, 2a, and 2b at 1 month were 53.1% and 87.5%, 31.5% and 94.7%, and 30.9% and 83.3%, respectively.

**Table 2:** Prognostic factors for survival at baseline in all patients (Cox hazard analysis)

	Univariate analysis			Multivariate analysis		
	HR	95% CI	*P*‐value	HR	95% CI	*P*‐value
Elderly (≥65 years old)	1.168	0.430‐3.168	0.761	–	–	–
Gender (female)	0.305	0.072‐1.302	0.109	–	–	–
BMI (≥21 kg/m^2^)	0.878	0.379‐2.035	0.762	–	–	–
TNM stage IV of LCSGJ 6th	1.493	0.338‐6.587	0.597	–	–	–
Positive for major portal vein invasion	2.489	0.732‐8.468	0.144	–	–	–
Alpha‐fetoprotein (>100 ng/mL)	0.970	0.419‐2.250	0.944	–	–	–
Positive for past history of TKIs	1.281	0.547‐3.001	0.569	–	–	–
Past history of HT	1.094	0.428‐2.801	0.851	–	–	–
Positive for DM	0.737	0.311‐1.747	0.488	–	–	–
Child‐Pugh score ≥ 7	4.998	1.789‐13.96	0.002	2.543	0.878‐7.364	0.085
mALBI 2b or 3	5.520	2.042‐14.92	<0.001	4.632	1.649‐13.02	0.004

– not applicable.

Abbreviations: HR: hazard ratio, CI: confidence index, BMI: body mass index, TNM of LCSGJ: tumor node metastasis stage of Liver Cancer Study group of Japan 6th edition, HT: hypertension, DM: diabetes mellitus, mALBI: modified albumin‐bilirubin grade

**Figure 4:**
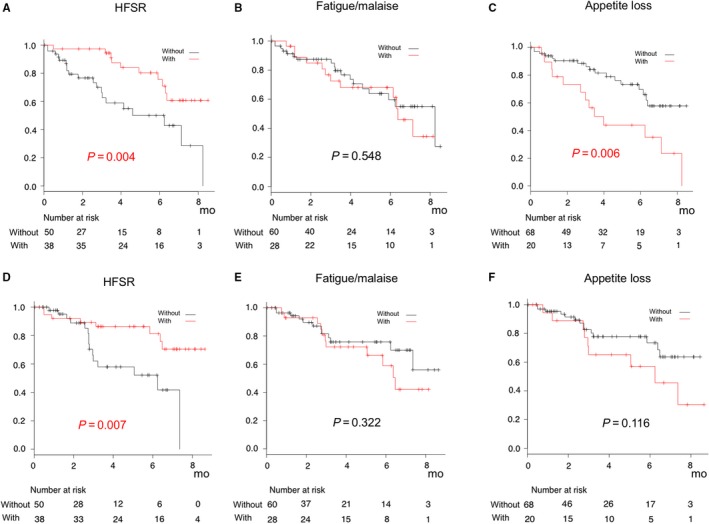
Time to stopping lenvatinib and time to progression (TTP) in patients with/without hand‐foot skin reaction, fatigue/malaise or appetite loss. (A‐C) Hand‐foot skin reaction (HFSR) (any grades) was negatively associated and appetite loss (any grades) positively associated with time to stopping LEN (*P* = 0.004 and *P* = 0.006, respectively), while fatigue/malaise was not (*P* = 0.548). (D) Estimated median TTP was significantly longer for patients with as compared to those without HFSR (no reached vs 8.9 months, *P* = 0.007), while (E, F) fatigue/malaise and appetite loss were not associated with TTP (*P* = 0.322 and *P* = 0.116).

Profiles of AEs, observed in 15% or more of all patients, are shown in Table 3. In patients with better hepatic reserve function (mALBI 1 and 2a), which is known to be associated with better prognosis, hand‐foot skin reaction (HFSR) (any grade) was negatively associated and appetite loss (any grade) was positively associated with time before stopping LEN (*P* = 0.004 and *P* = 0.006, respectively), while fatigue/malaise was not (*P* = 0.548) (Figure 4A‐C). The BMI of patients with appetite loss (any grade) was lower as compared to those without that AE (20.3 ± 3.0 vs 23.6 ± 4.0 kg/m^2^, *P* < 0.001), while the cut‐off value for predicting appetite loss shown by the ROC method was 20.7 kg/m^2^ (sensitivity 0.797, specificity :0.632, area under the curve 0.726, 95% CI 0.594‐0.858). The TTP of patients with HFSR was significantly longer as compared to those without that AE (estimated median TTP: not reached vs 8.9 months, *P* = 0.007), while fatigue/malaise and appetite loss were not associated with TTP (*P* = 0.322 and *P* = 0.116) (Figure 4D‐F). For patients with HFSR, ORR and DCR at 1 month after starting LEN were 59.1% and 86.4%, respectively.

**Table 3:** Adverse events observed in 15% or more of all patients (n = 152) and patients with good hepatic function (n = 88)

	All (n = 152)			Good hepatic function (mALBI 1 and 2a) (n = 88)		
	Grade 1/2	Grade 3/4	All Grades	Grade 1/2	Grade 3/4	All Grades
HFSR	39	8	47 (30.9%)	32	5	37 (57.9%)
Fatigue/malaise	37	9	46 (30.2%)	21	7	28 (31.2%)
Appetite loss	26	9	35 (23.0%)	17	3	20 (22.7%)
Diarrhea	25	5	30 (19.7%)	14	3	17 (19.3%)
Thyroid function abnormality	28	2	30 (19.7%)	20	1	21 (23.8%)
Hypertension	23	5	28 (18.4%)	15	2	17 (19.3%)
Urine protein	14	9	23 (15.1%)	10	8	18 (20.5%)

Abbreviations: mALBI: modified albumin‐bilirubin grade, HFSR: hand‐foot skin reaction

## 4 DISCUSSION

It is well known that the prognosis of HCC patients is dependent on tumor burden and hepatic reserve function.^13^, ^25^, ^26^ Especially for those who undergo TKI treatment, hepatic function has been shown to be the most important prognostic factor.^27^, ^28^, ^29^ Although Child‐Pugh score and class are used for assessment of hepatic function worldwide,^9^ they have been shown to be problematic when calculated using semiquantitative scoring, and also known to include both confounding (eg, albumin, ascites) and nonobjective (ascites, hepatic encephalopathy) factors. Recently, ALBI, calculated with only two factors, has been developed as a suitable statistical tool for assessment and shown to provide a more detailed evaluation of hepatic reserve function.^12^, ^13^, ^14^ In patients with SOR treatment, ALBI 1 was reported to be a better prognostic factor than ALBI 2, even in patients with a Child‐Pugh score of 5.^28^ On the other hand, Child‐Pugh class and ALBI have a common weak point, in that the intermediate grade of each (Child‐Pugh class B, ALBI grade 2) covers a very wide range. Ogasawara et al^27^ subdivided ALBI grade 2 based on median score and found that the better one indicated longer OS for patients treated with SOR. To provide a more detailed assessment of hepatic reserve function, mALBI with four grades has been proposed by dividing ALBI grade 2 into 2a and 2b statistically using an ALBI score of −2.270 for the cut‐off, which was shown to be the predictive value for 30% of ICG‐R15.^10^, ^11^

Although past history of TKI was not a prognostic factor in the present analysis for the prognostic factors at baseline, therapeutic effect of LEN might be worse in patients with PD than the others (non‐PD) with regard to therapeutic best‐response of previous SOR treatment (*P* = 0.012). More accumulation of clinical data of patients will be needed from the view of therapeutic efficacy of past TKI treatment. We found a much lower therapeutic efficacy of LEN in patients with Child‐Pugh grade B as compared to A, thus that treatment in Child‐Pugh B patients should be performed discreetly and with caution. Moreover, patients with mALBI 2b or 3, scores that were stronger for poor prognosis than Child‐Pugh score of 7 or more, showed worse prognosis than the other patients, even those classified as Child‐Pugh A. Based on the present findings, we consider that the best indication for LEN in a patient with u‐HCC is good hepatic function, such as mALBI 1 or 2a. Our results showed that hepatic function at the start of therapy was the only important prognostic factor related to LEN, thus appropriate judgment with regard to transcatheter arterial chemoembolization (TACE) failure has become an important issue for maintaining hepatic function in this multi‐TKI era.

In the present cohort, therapeutic response at 1 month after starting LEN (ORR/DCR) was similar to results obtained in the REFLECT trial.^6^ Patients with PD at 1 month showed worse prognosis than the others (*P* < 0.001). Although therapeutic response was an important predictor for prognosis after starting LEN, and the rates of ORR and DCR in patients with mALBI 2a (31.5% vs 30.9%) and 2b (94.7% vs 83.3%) were similar, the prognosis of mALBI 2b cases was worse as compared to mALBI 2a. This finding indicates that hepatic function at the start of therapy is a more important prognostic factor regarding LEN treatment, which shows a high DCR.

The frequency of each AE (all grades) in all patients was similar to that of patients with good hepatic function (mALBI 1, 2a), except for HFSR. HFSR is thought to be a predictor of good therapeutic response in patients receiving LEN treatment as well as those treated with SOR.^30^ On the other hand, appetite loss can cause stoppage of LEN and the BMI of patients with that AE was significantly lower (*P* < 0.001). Porta et al reported that a low dose and treatment discontinuations were correlated with worse survival in renal cell carcinoma patients treated with sunitinib, indicating the importance of maintaining dose intensity.^31^ In a previous study of LEN treatment for thyroid cancer, progression‐free survival was shorter in patients with a longer dose‐interruption period.^32^ Although lower BMI (<21.0 kg/m^2^) was not associated with poor prognosis in the present observation period (Table 3), maintaining quality of life (QOL) and avoiding dose‐interruption, which is associated with reducing the amount of administration of LEN, are important in chronic liver disease patients treated with LEN. When LEN is given to patients with lower BMI (<21.0 kg/m^2^), close attention is needed for decline of appetite, while a dose adjustment may be required soon in accordance with the condition in order to avoid a long period of interruption or stopping the drug, which can reduce therapeutic efficacy.

Although this was a multicenter study, a limitation of the study is its retrospective nature. Furthermore, a longer observation period and comparison with other therapeutic modalities in real‐world practice are needed to obtain more concrete conclusions. Nevertheless, we found that hepatic reserve function was the only prognostic factor related to survival in our patients treated with LEN. Assessment based on mALBI was found useful for a detailed evaluation of hepatic function as compared to Child‐Pugh score. Patients with good hepatic function, such as mALBI 1 and 2a, have the best indication for LEN, though appetite loss must be kept in mind in those with low BMI (<21 kg/m^2^), regardless of mALBI score.
