# Supplementary figures and images for: Prognostic factor of lenvatinib for unresectable hepatocellular carcinoma in real‐world conditions—Multicenter analysis
Source: Cancer Med. 2019 May 24;8(8):3719–28. doi: 10.1002/cam4.2241 (PMC6639201; doi:10.1002/cam4.2241)

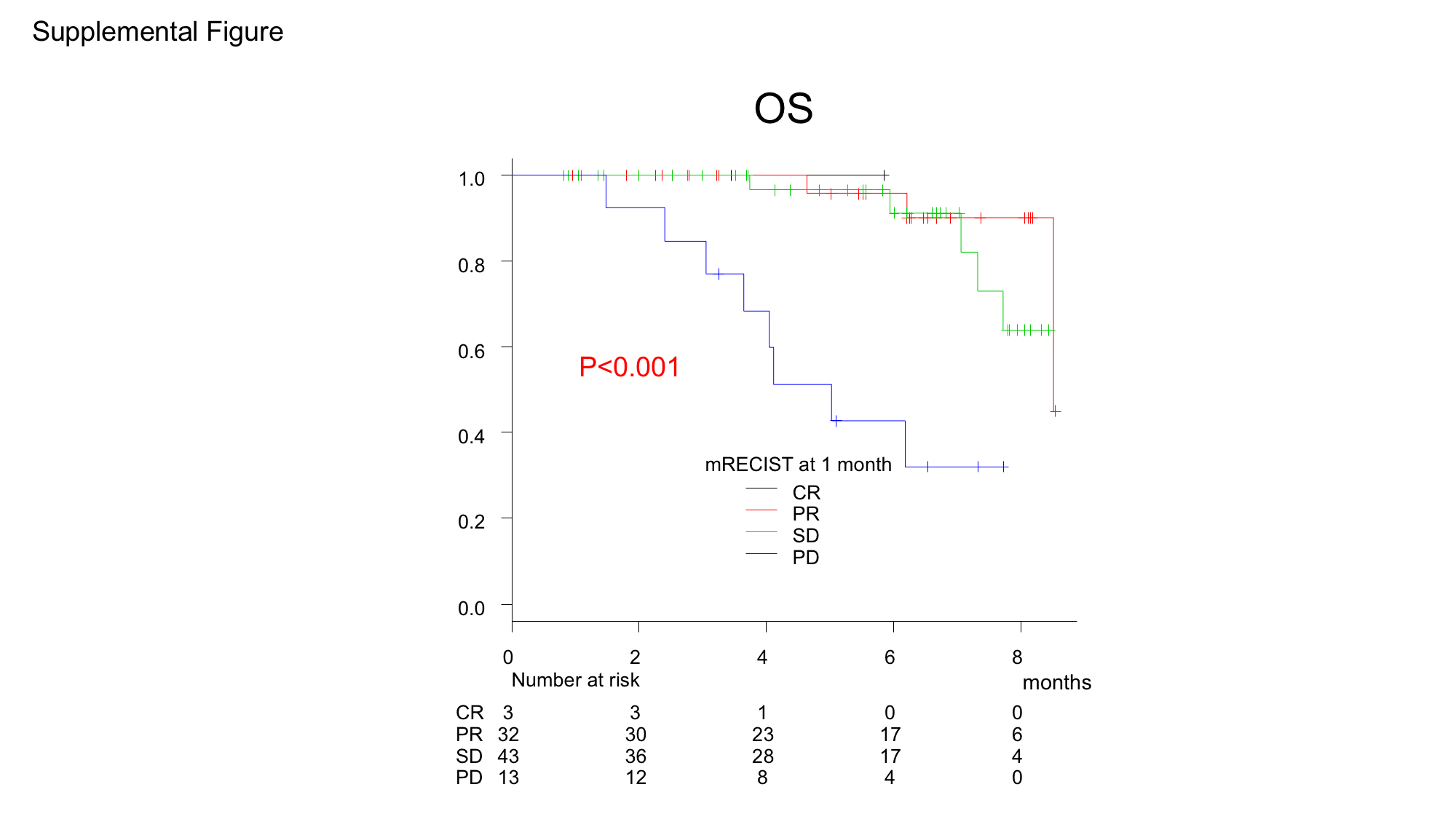

Supplement: Supplementary file 1 [file CAM4-8-3719-s001.tiff]

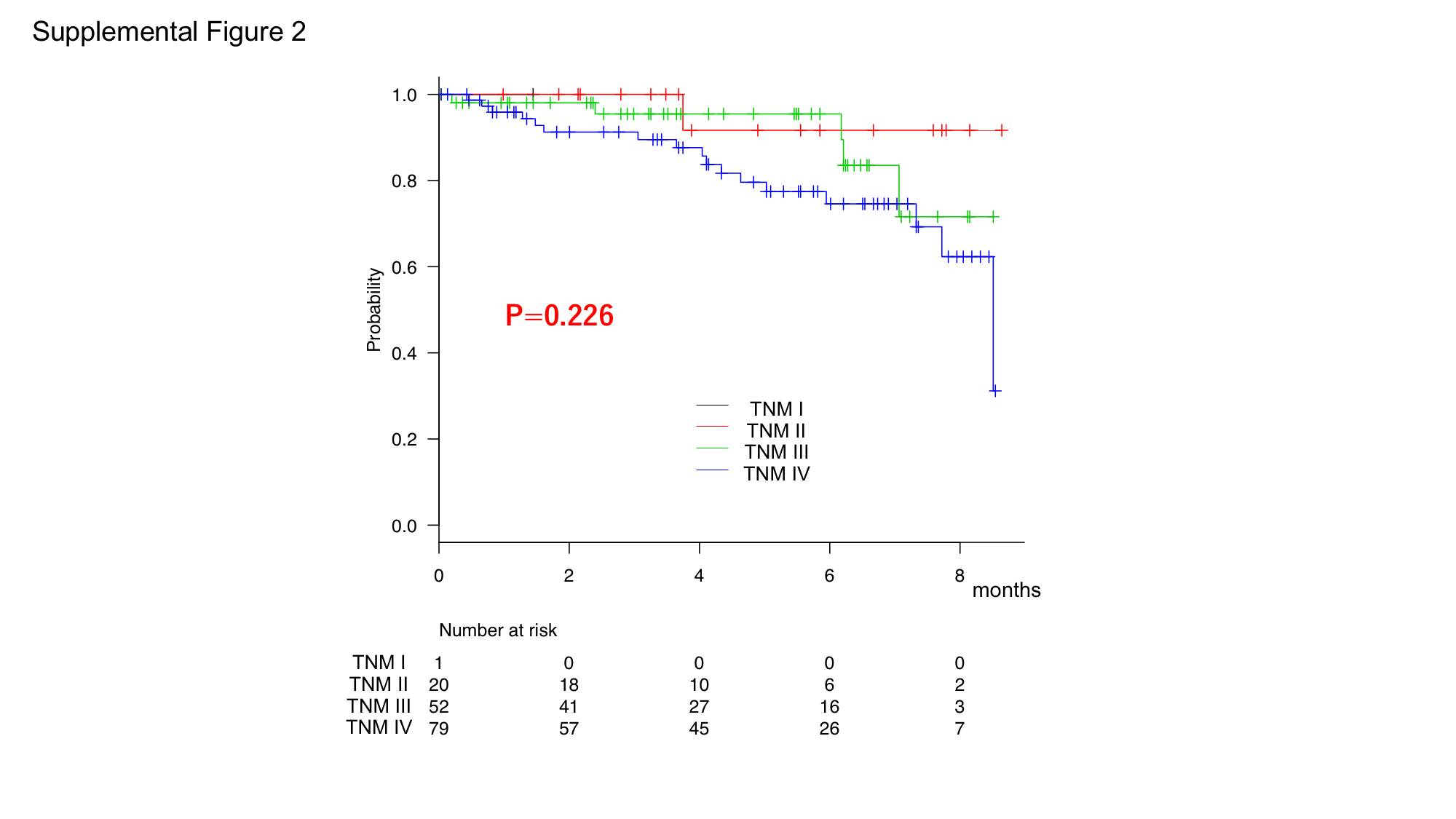

Supplement: Supplementary file 2 [file CAM4-8-3719-s002.tiff]

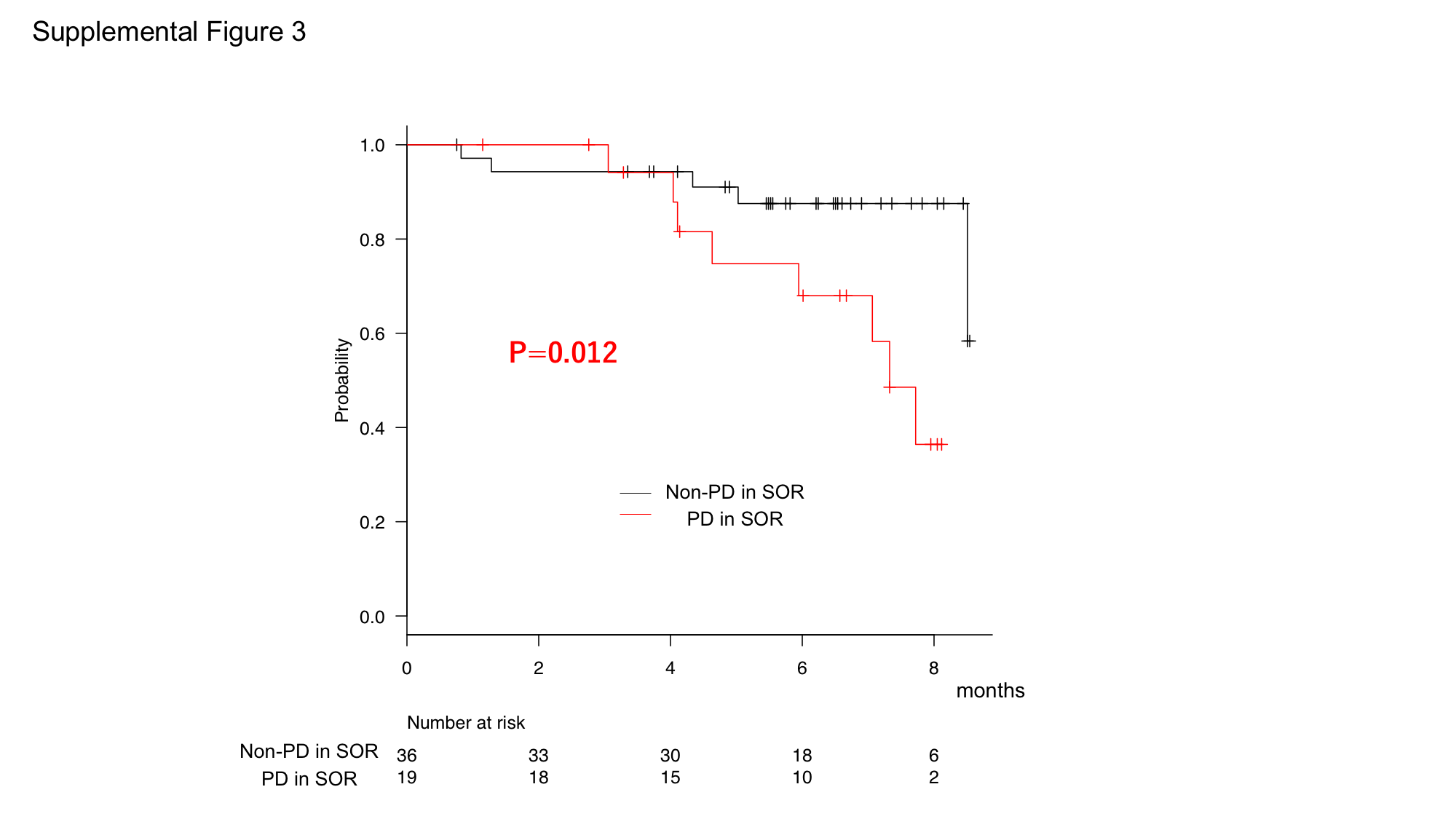

Supplement: Supplementary file 3 [file CAM4-8-3719-s003.tiff]
